# Pet fur or fake fur? A forensic approach

**DOI:** 10.1186/2041-2223-5-7

**Published:** 2014-06-02

**Authors:** Elena Pilli, Rosario Casamassima, Stefania Vai, Antonino Virgili, Filippo Barni, Giancarlo D’Errico, Andrea Berti, Giampietro Lago, David Caramelli

**Affiliations:** 1 Dipartimento di Biologia, Università di Firenze, via del Proconsolo 12, 50122 Florence, Italy; 2 Reparto Carabinieri Investigazioni Scientifiche di Roma, Sezione di Chimica, viale Tor di Quinto 119, Rome, Italy; 3 Istituto Superiore di Tecniche Investigative dei Carabinieri, viale Salvo D’Acquisto 2, 00049 Velletri (Roma), Italy; 4 Reparto Carabinieri Investigazioni Scientifiche di Roma, Sezione di Biologia, viale Tor di Quinto 119, Rome, Italy; 5 Reparto Carabinieri Investigazioni Scientifiche di Parma, Parco Ducale 3, Parma, Italy

**Keywords:** Fur samples, High degraded samples, Microscopic analysis, mtDNA, Species identification

## Abstract

**Background:**

In forensic science there are many types of crime that involve animals. Therefore, the identification of the species has become an essential investigative tool. The exhibits obtained from such offences are very often a challenge for forensic experts. Indeed, most biological materials are traces, hair or tanned fur. With hair samples, a common forensic approach should proceed from morphological and structural microscopic examination to DNA analysis. However, the microscopy of hair requires a lot of experience and a suitable comparative database to be able to recognize with a high degree of accuracy that a sample comes from a particular species and then to determine whether it is a protected one. DNA analysis offers the best opportunity to answer the question, ‘What species is this?’ In our work, we analyzed different samples of fur coming from China used to make hats and collars. Initially, the samples were examined under a microscope, then the mitochondrial DNA was tested for species identification. For this purpose, the genetic markers used were the *12S* and *16S* ribosomal RNA, while the hypervariable segment I of the control region was analyzed afterwards, to determine whether samples belonged to the same individual.

**Results:**

Microscopic examination showed that the fibres were of animal origin, although it was difficult to determine with a high degree of confidence which species they belonged to and if they came from a protected species. Therefore, DNA analysis was essential to try to clarify the species of these fur samples.

**Conclusions:**

Macroscopic and microscopic analysis confirmed the hypothesis regarding the analyzed hair belonging to real animals, although it failed to prove with any kind of certainty which actual family it came from, therefore, the species remains unknown. Sequence data analysis and comparisons with the samples available in GenBank showed that the hair, in most cases, belonged to the *Canidae* family, and in one case only to *Felidae*.

## Background

Forensic analysis tends to focus mainly on human DNA typing, but this is not the only source of DNA that can be found at a crime scene. Many types of crime involve nonhuman DNA samples, such as those of animals, plants, pollen, bacterial and viruses [1-4]. Despite being a relatively new subdiscipline of forensic science, the interest of the forensic community regarding nonhuman DNA samples is considerable, as evidenced by [5]. The identification of a species and the linking of a sample to a specific individual are becoming important tools in forensic investigation, owing to an increase in wildlife crime. One of the main reasons for the increase of wildlife crime probably comes from the fact that it is highly profitable and the penalties for guilty people are often light. Most of the time, the potential profit far outweighs the maximum penalty for the alleged crime [6]. Interpol estimates that illegal wildlife trade is the largest black market worldwide, second only to the traffic in drugs [7-9].

Many mammalian species, such as tigers, elephants, bears, rhinoceroses, and, mainly, reptiles and amphibians are trafficked every year around the world [10-19]. Crimes range from the abuse of illegal trade in protected species, or parts of them, such as horns, eggs [20] and tusks used to make ornaments or jewellery [21-26] or fur used to make gloves, hats and collars, to poaching and hunting out of season and many others. Italy was the first in Europe, and second in the world after the USA, to have imposed a ban on imports and the domestic trade of skins and furs from protected animals, firstly, through the ordinances of Dr Sirchia, the Minister of Health, issued in 2001 and renewed within two years, and then, in 2004, with the inclusion of the prohibition into the law against the mistreatment of animals [27]. The Italian law (article 2) prohibits the use of skins and furs for commercial purposes: ‘Dogs (*Canis familiaris*) and cats (*Felis catus*) may not be used in the production or packaging of skins, furs, clothing and leather goods made or produced, in whole or in part, from the their skins or fur, as well as their skins or fur, cannot be marked or introduced into the national territory. Violation shall be punishable by imprisonment for periods of three months to one year or by a fine from 5,000 to 100,000 euros’. Therefore, the ability to identify a particular species and the capacity to determine whether the biological sample belongs to a specific individual of that species are an important tool in investigations.

Morphologic and structural microscopic analyses are the first approach in investigating the nature of the samples, excluding synthetic origins of the fibres, distinguishing between human and animal hair and trying to identify the species. These techniques are also important in comparing the features of the questioned hair with those collected from a known individual. In the case of animal hair, it is important to focus on shapes and sizes, colour banding, cuticular patterns, medulla organization, root structure and scales for its identification. A table presented in the *Encyclopedia of Forensic Science*[28] shows in detail all animal hair characteristics and traits that have to be considered during microscopic examination. Moreover, an adequate reference collection is essential for accurate identification of samples. Indeed, the wide variation in characteristics of hair taken from different body areas on the same animal and the large differences among animals that can occur within the same species do not allow the identification of the species of animal hair if a complete and accurate reference sample of hair is not available. Furthermore, much experience in microscopic hair analysis is required before one can confirm with a high degree of confidence whether or not a sample belongs to a protected species. However, even with experience, sometimes only the taxonomic rank of family may be identified.

When species identification using these methods is inconclusive, the only possible solution is to resort to more expensive DNA analysis. The application of DNA-based technologies to species identification has made the investigation of wildlife crimes possible. For several reasons, the DNA markers used in forensic species identification are located in the mitochondrial genome and derived from taxonomic and phylogenetic studies [29]. One of the main reasons for choosing mitochondrial DNA (mtDNA) as a forensic marker in identification studies is that there is no recombination. With the exception of mutations, all the mtDNA molecules of an individual are equal and inherited through the maternal line [30,31]. Moreover, the mtDNA molecules are present in multiple copies per cell [32] and the mitochondrion has a protein coat that helps to protect it from degradation: in highly degraded samples, such as bones, teeth and, obviously, the hair shaft [33-39], these characteristics make this approach more suitable than the nuclear DNA approach. Additionally, enzymes in the mitochondria are not able to read and correct DNA bases added incorrectly during replication [40], so that mutations can be accumulated in mtDNA up to five times more quickly than those accumulated during nuclear DNA replication.

There are many studies of species identification and many different markers can be utilized. The choice of marker for forensic species identification shows little intraspecific variability, that is, variability between members of the same species, but demonstrates sufficient interspecific variability, that is, between members of different species, to discriminate between individuals of different species. One of these loci is *cytochrome b*[41-43]. Over the years, several forensic and taxonomic studies have utilized *cytochrome b* as a marker for species identification [44-56]. However, more recently, there has been an increase in the use of another marker called *cytochrome c oxidase I* (*COI*). This is used in DNA barcoding; it has been adopted as a marker by the Barcode for Life Consortium [57-60]. Initially, it was used in the identification of invertebrate species [61-65]; for example, it has been exploited as a marker in forensic entomology to identify the beetle larvae on a corpse [66,67]. Since then, *COI* has been proposed for the identification of many organisms [68-82]. Other mitochondrial genes can be utilized as markers in forensic species identification: *12S* ribosomal RNA [83-85], *16S* ribosomal RNA [86-88] and the *NDH* family [89,90]. While all these genes have been investigated for species identification, the D-loop analysis has mostly been exploited for intraspecies identification [91-96], though sometimes it can be applied to species identification [23,43,85,97-99]. Usually, the standard analysis of species identification requires amplification of part of these genes, followed by sequencing and comparison of the obtained sequence with reference sequences kept in a database, such as GenBank.

Here, we report a case of the alleged illegal trade of a protected species of fur. Our work consisted of ascertaining whether the furs had a synthetic origin or belonged to *Canis lupus familiaris*, *Felis silvestris catus* or other species. Furthermore, we had to assess whether the different furs belonged to the same individual. Initially, we focused on ruling out a synthetic origin for the fur and on trying to assess which species the hair belonged to by microscopic analysis. Finally, the species was identified by DNA analysis. The genetic markers used were *12S* and *16S* rRNA and the control region (hypervariable segment I, HVS-I) for individual identification. The advantages of this assay were: (1) the abundance of the mtDNA genome, (2) the broad reactivity of the primers that permitted the amplification of the DNA from unknown samples, (3) the small size of the amplicons, particularly the *12S* rRNA, which was ≈ 150 base pairs (bp) and (4) the amplification of seven overlapping HVS-I fragments with sizes ranging from 130 to 250 bp.

## Methods

### Samples

Twelve fur samples (named A1, A2, A3, A4, A5, B1, B2, B3, B4, B5, B6 and B7) of alleged animal origin from protected species, in Italy, coming from China and used to make hats and collars, were seized just outside Genoa. In Italy, since the law 189/2004 has prohibited use of the skins and furs of domestic dogs (*Canis lupus familiaris*) and cats (*Felis silvestris catus*) for commercial purposes, microscopic and DNA evidence was necessary to ascertain the origin of these fibres and permit identification of the species. The seized fur was of different colours: black, brown, grey and red. Despite being subjected to thermal, chemical and mechanical stress during the processing cycle of tanning, the furs showed an apparently good state of preservation. However the amount of DNA in hair [100] is low compared with other tissues. Indeed, hair cells undergo dehydration and catabolic breakdown of nucleic acids and organelles during keratinization. Nevertheless, forensic scientists have successfully amplified DNA from modern human hair [101] and analyzed mtDNA from degraded and old hair samples [102-105].

### Microscopic analysis

#### *Stereomicroscopy*

First, the samples were carefully observed using a Leica M 651 stereomicroscope (Leica Microsystems Srl, Milano, Italy). This allowed the observation of samples in three dimensions, thereby avoiding the flattening effect typical of other microscopes. At low magnifications of approximately 6.5× to 40×, this analysis permitted the reproduction a three-dimensional image in direct vision and the examination of colour, texture, structure and treatment effects of samples.

#### *Light microscopy*

In forensic laboratories, the examination of the internal structures of this type of sample is typically carried out using a light microscope. Therefore, the hair samples were prepared for microscopic examination by placing them on glass slides with a colourless mounting medium in the refractive index range of 1.5 to 1.60. The mounting medium utilized was Hi-MO (Bio Optica, Milano S.p.A., Italy) with a refractive index of 1.5. To see a cuticular pattern more clearly, it was necessary to make a scale cast of the hair specimens. A thin coating of clear nail polish was set on a microscope slide. While still wet, each hair was positioned in the nail polish, ensuring that the entire length of the hair was included. Then, when the nail polish was completely dry, the hair was gently removed to obtain the scale cast [106]. To prevent contamination of each fur sample, the microscope slides were prepared under a hood. The light microscope utilized was a Leica DM LB2 (Leica Microsystems Srl, Milano, Italy).

### Genetic analysis

#### *Highly degraded DNA analysis*

To obtain reliable results, owing to the stress caused by the tanning process and the characteristics of the hair, genetic analysis for these findings was conducted following the most stringent criteria proposed for ancient DNA studies [107-109]. Accepted authentication methods must be employed to assess the authenticity of the results. Different precautions were taken to avoid contamination of samples with extraneous DNA: (1) all DNA extraction and PCR involving the samples was carried out in a laboratory physically separated from the laboratory in which PCR cycling and post-PCR analyses were performed; (2) disposable masks, gloves and laboratory coats were worn throughout and were changed frequently; and (3) all DNA extractions and PCR reactions included negative controls. Repeated amplification from the same or different extracts from the same specimens are necessary: (1) to identify contamination of a particular extraction or amplification; (2) to allow DNA analysis when the extracts only sporadically contain DNA template molecules; and (3) to detect nucleotide misincorporations that produce consistent changes in the DNA molecule. In highly degraded samples, one of the most common modifications of (or damage caused to) the DNA molecule is the hydrolytic loss of amino groups from the nitrous bases [110]. The deamination products of cytosine (uracil), 5-methylcytosine (thymine) and adenine (hypoxanthine) have particular relevance to the amplification of ancient DNA, since they cause the incorrect bases (A instead of G and C instead of T) to be inserted when new DNA strands are synthesized by a DNA polymerase [111,112]. In addition, ‘jumping PCR’ , that is, the occurrence of template switching during PCR, may contribute to these substitutions. To distinguish between these two types of miscoding lesion, multiple amplifications of DNA extracts were performed and the PCR products were cloned. By comparing different clones from multiple amplifications, it will be possible to highlight the nucleotide differences that occur in all clones of one amplification from those that occur only sporadically [113]. A consensus sequence can be constructed from the selected clone sequences once data from possible jumping PCR events has been identified and removed. Bower *et al.*[114] demonstrated that 20 clones must be sampled from a single forensic or ancient DNA sample in order to determine a reliable consensus sequence.

DNA fragments derived from genomes of organelles, such as mitochondria [115], are often present in the nuclear genome [116] and, as reported by Jan den Tex *et al.*[117], they cause another problem for ancient DNA analysis. Since mitochondrial DNA is the molecule of interest in most ancient DNA projects, such nuclear integrations may occasionally be amplified by PCR and be mistaken for the organellar DNA sequences. This is particularly likely to happen if the primers used differ from the organellar DNA sequence in the individual specimen but not from the version of the same sequence that exists as a nuclear insertion. Erroneous conclusions regarding intraspecific variation [118], as well as species phylogenies [119], will then result. To prevent this problem, different primer sets can be used to amplify different overlapping fragments covering a target region, since it is very unlikely that two or more primer sets would preferentially amplify a particular nuclear insertion [119,120]. Furthermore, the use of multiple genes improves the chances of correctly identifying the species of the sample [121,122].

#### *Sample preparation and DNA extraction*

Sample preparation, DNA extraction and PCR reaction setup were carried out in a laboratory dedicated to ancient DNA analysis. The pre-PCR area was physically separated from the area in which PCR cycling and post-PCR analyses were conducted. The reagents, bench surface and nondisposable equipment were sterilized under ultraviolet light. Four or five hairs from each fur sample were decontaminated by soaking in a 5% bleach solution for 30 s, followed by a rinse carried out initially in absolute ethanol and then in sterile distilled water. The samples were left to air-dry and then cut into small pieces, about 2 to 3 mm, and collected in a sterile 1.5 ml vial. The DNA extraction was performed according to the protocol of Gilbert *et al.*[105]. The fragmented samples were digested overnight at 55°C in a 0.5 ml extraction buffer (modified, as to the protocol of Barnes *et al.*[123]) containing 0.01 M Tris buffer, 0.01 M NaCl solution, 1% SDS, 0.5 mg/ml proteinase K, 10 mg/ml dithiothreitol and 0.001 M *N*-phenacylthiazolium bromide. The DNA was extracted using a phenol-chloroform DNA extraction protocol, purified and concentrated by Amicon ultra 0.5 ml centrifugal filters (Millipore), according to the user’s manual, to yield a final volume of approximately 50 μl. At least two independent extracts were obtained from each fur sample. Negative controls were included in each extraction set.

#### *PCR amplification of 12S and 16S*

Two microlitres of DNA from each extract were amplified as follows: 94°C for 10 min (Taq polymerase activation), followed by 40 cycles of PCR (denaturation, 94°C for 45 s, annealing, 53°C for 1 min and extension, 72°C for 1 min) and then a final step at 72°C for 10 min. The 50 μl reaction mix contained 2 U of AmpliTaq Gold (Applied Biosystems), 200 μM of each deoxyribonucleotide triphosphate (dNTP), 1.5 mM MgCl_2_, 1× PCR buffer and 1 μM of each primer. According to Melton *et al.*[84], the *12S* primer sequences amplified a 150 bp fragment. According to Kitano *et al.*[85], the *16S* primer sequences amplified a 244 bp fragment. Each extract was amplified in duplicate. The extraction reagent control, a known positive control (human DNA) and a negative PCR control were amplified in parallel. Thirty-five microlitres of each PCR product were analyzed by electrophoresis, on a 1.5% agarose gel for 45 min at 80 V and visualized with ethidium bromide. To identify the molecular-weight size of PCR products we used a 50 bp ladder (New England BioLab Inc.). The PCR products were purified using a Montage™ DNA gel extraction kit (Millipore), according to the user’s manual.

#### *PCR amplification of dog HVS-I*

The control region of dog mtDNA is 1270 bp long and contains two hypervariable segments (HVS-I and HVS-II) and a 10 nt variable number tandem repeat (VNTR) between HVS-I and HVS-II [95]. We analyzed the HVS-I region and, as recommended for analysis of ancient DNA samples, the hypervariable region was divided into overlapping fragments, to prevent the amplification of nuclear insertion and increase the probability of amplification success if the DNA is degraded and fragmented [119,120]. The 672-bp-long HVS-I was subdivided into seven overlapping fragments, using the primer pairs reported in Table 1. Some primer sequences, such as L15422/H15555 and L15511/H15692, utilized for analysis, were obtained from Verginelli *et al.*[124,125]*.* The other primers were designed using the Primer3 program [126]*.* The nomenclature of the primers corresponds with the 5′-end of the reference sequence, according to [127]*.* The size of PCR products ranged from 130 to 250 bp. The HVS-I was amplified in a final volume of 50 μl in seven different assays including 2 U of AmpliTaq Gold (Applied Biosystems), 200 μM of each dNTP, 1.5 mM MgCl_2_, 1× PCR buffer and 1 μM of each primer. Each fragment was amplified in duplicate. The extraction reagent control, a known positive control (dog DNA) and a negative PCR control were amplified in parallel. The PCR products were analyzed by electrophoresis on a 1.5% agarose gel, as described in the previous paragraph.

**Table 1:** Primer pairs used to amplify the seven overlapping fragments of HVS-I with molecular details

**Amplicon size (bp)**	**Primer name**	**Primer range**	**Primer sequence 5′-3′**	**Sequence information range (bp)**	**Reference**
**170**	L15422 H15555	15404-15422 15555-15574	CTCTTGCTCCACCATCAGC TTATATGCATGGGGCAAACC	133	[124]
**219**	L15511 H15692	15490-15511 15692-15709	ACTGTGCTATGTCAGTATCTCC TTGATGGTTTCTCGAGGC	181	[124]
**246**	L15574 H15780	15555-15574 15780-15801	GGTTTGCCCCATGCATATAA AAGTAAGAACCAGATGCCAGGT	206	[126]
**148**	L15764 H15873	15744-15764 15873-15892	CCCATACTAACGTGGGGGTTA TGTGTGATCATGGGCTGATT	109	[126]
**129**	L15857 H15945	15836-15857 15945-15965	ATTCTCGCAAATGGGACATC GCGGTCGTAGGTGAGTGATAG	88	[126]
**137**	L15892 H15991	15873-15892 15991-16010	AATCAGCCCATGATCACACA GGCATATTCCCTGAGAGCAG	99	[126]
**154**	L15982 H16097	15963-15982 16097-16117	CGCAACGGCACTAACTCTAA TGTCCTGAAACCATTGACTGA	115	[126]

#### *Cloning and sequencing*

The PCR products were cloned using a TOPO TA Cloning Kit (Invitrogen), according to the manufacturer’s instructions. Screening of 20 white recombinant colonies was accomplished by PCR, and the colonies were transferred into a 30 ml reaction mix (67 mM Tris HCl (pH 8.8), 2 mM MgCl_2_, 1 mM of each primer, 0.125 mM of each dNTP, 0.75 units of Taq polymerase) containing M13 forward and reverse universal primers. After 5 min at 92°C, 30 cycles of PCR (30 s at 90°C, 1 min at 50°C, 1 min at 72°C) were carried out and clones with samples of the expected size were identified by agarose gel electrophoresis. After the purification of all PCR products, using a PureLink™ PCR Purification kit (Invitrogen™), a volume of 1 μl was cycle-sequenced using a BigDye® Terminator v3.1 cycle sequencing kit™ (Applied Biosystems), following the supplier’s instructions. Sequencing involves initial denaturation at 96°C for 1 min followed by 25 cycles at 96°C for 10 s, 50°C for 5 s and 60°C for 4 min. Sequencing reaction products were purified from residual dye terminators using Agencourt® CleanSEQ® (Beckman Coulter), according to the manufacturer’s manual. Sequencing was carried out on Applied BioSystems 3130 × l Genetic Analyzer (Applied Biosystems) using POP7, 36 cm capillary arrays and the default instrument settings, as recommended by the manufacturer. The data were analyzed using SeqScape® Software Version 2.5 (Applied Biosystems) and default software settings, as recommended by the manufacturer. Twenty clones for each sample were sequenced, and the sequences obtained were aligned using the Clustal X program [128] and compared across clones. Then, for each sample, the consensus sequence of *12S*, *16S* and HVS-I was copied and pasted into the nucleotide-nucleotide BLAST function at the National Centre for Biotechnology Information (NCBI) website [129]*.* The BLAST portal generated a report showing the degree of sequence similarity with organisms in the NCBI database. If no NCBI reference presented a similarity of 100% with all the query sequences, further phylogenetic analyses were performed. Species identifications were made based on the percentage similarity of the query sequence to the reference sequence (>98%) as described in previous papers [20,130].

#### *Phylogenetic analysis*

A comparison dataset of sequences of the mitochondrial HVS-I was compiled, in order to represent the known intra- and interspecific variation between the species and subspecies for which almost 98% of similarity was detected by the nucleotide-nucleotide BLAST search with the results obtained from the fur samples. All the sequences (from the dataset and the fur samples) were analyzed using MEGA version 6 [131]; they were aligned using the MUSCLE algorithm [132], and the best substitution model was assessed and used to develop a neighbour-joining tree [133], including a bootstrap analysis with 1,000 replications, to determine which species or subspecies the fur samples clustered with.

## Results and discussion

### Macroscopic and microscopic analysis

**Figure 1:**
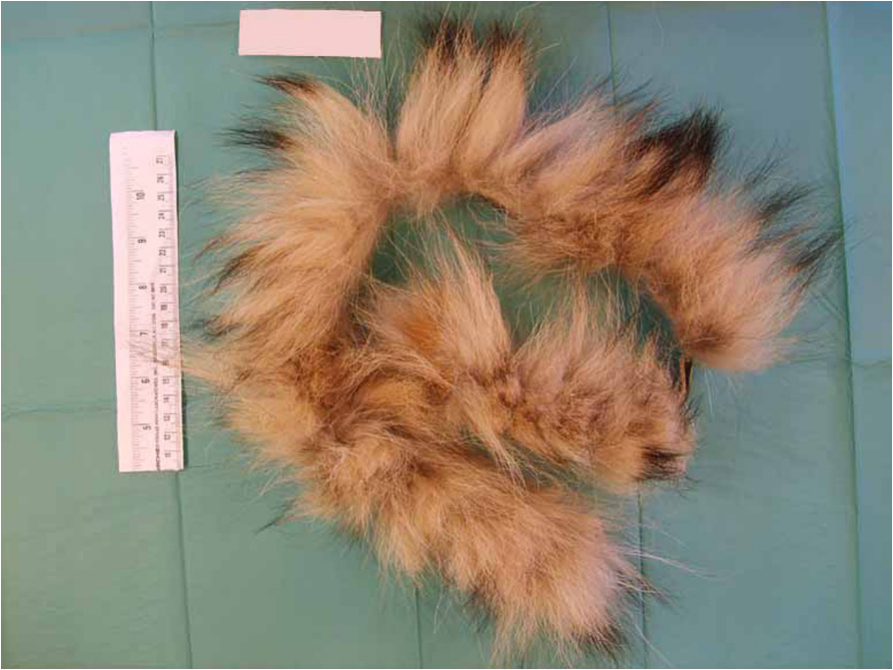
**Sample A2.** Example of colour changing over relatively short sections of the shaft of the sample.

**Figure 2:**
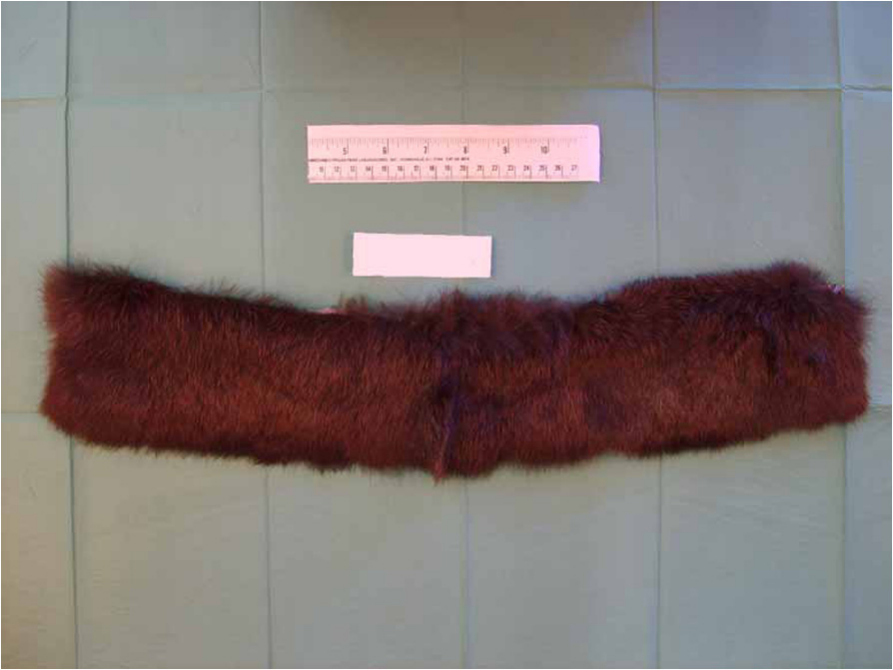
**Sample B7.** Example of the artificial, roughly homogeneous colour of the sample.

The analysis of hair samples began with a macroscopic approach and then moved to a microscopic one. The visible characteristics of the samples were initially observed with the naked eye and described, and then studied using a stereomicroscope (macroscopic analysis) to determine colour, texture, structure and treatment effects of the samples. The seized samples consisted of fur of different colours (Table 2).Some of the fur samples, such as A1, A2, A3, A4, A5, B5 and B6, were a natural colour, with the colour changing over short distances (banding) (Figure 1). Others, such as B1, B2, B3, B4 and B7 were of artificial colours, roughly homogeneous (Figure 2).

**Table 2:** Different colours of the seized furs

**Samples**	**Colour of fur**
A1	Banding: yellowish and black
A2	Banding: yellowish and black
A3	Banding: white and dark brown
A4	Banding: white and dark brown
A5	Banding: yellowish and black
B1	Red with dark shades
B2	Dark brown
B3	Brown
B4	Dark brown
B5	Banding: white and dark brown
B6	Banding: white and dark brown
B7	Dark brown

The natural colour banding, only present in some samples, is a typical characteristic of several animal species, such as the mink, muskrat, raccoon, otter and many others, but is generally not typical of the cat or the dog. The homogeneous colour of the other samples was unnatural and led us to believe that the furs were dyed. Careful examination also revealed the presence of both longer, thicker hair and shorter, thinner hair. The observed differences in length and texture were compatible with animal furs: in mammalian species, with the exclusion of human beings and cetaceans, the longest and thickest hairs are known as guard hairs, while the shortest and thinnest are known as wool hairs. The guard hair protects the animal from rain and snow, and determines the colour of the coat, while the wool hair represents the bottom layer of the coat and its main function is to keep the body temperature stable. Both guard and wool hair appeared heterogeneous in length, thickness and consistency. Moreover, a thorough inspection revealed that some of the hair seemed to originate from a single structure: this was probably due to complex hair follicles. The complex hair follicle, a typical structure of, for example, dog and cat hair, is an epidermal invagination in the dermis, whereby different hairs can grow from the same follicle. The internal structure of the hair was then investigated using a light microscope. Microscopic analysis showed the morphostructural characteristics of the hair. In particular, it confirmed the presence of three distinct layers from the outside inwards: the cuticle, the cortex and the medulla. The morphostructural characteristics observed for each hair are shown in Table 3.

**Table 3:** **Morphostructural characteristics of the seized furs**[134]

**Samples**	**Cuticle pattern**	**Cortex**	**Medulla pattern**	**Individual characteristics**
**A1**	Double chevron	Uniform	Opaque unbroken cellular	Cortex: more dense towards medulla
**A2**	Double chevron	Uniform	Opaque unbroken cellular	Root shape: spade-like
**A3**	Double chevron	Uniform	Opaque unbroken cellular	Cortex: more dense towards medulla
**A4**	Double chevron	Uniform	Opaque unbroken	Cortex: more dense towards medulla
**A5**	Double chevron	Uniform	Vacuolated	Cortex: more dense towards medulla
**B1**	Double chevron	Uniform	Vacuolated	
**B2**	Regular petal	Uniform	Opaque unbroken	Medulla pattern: wide
**B3**	Double chevron	Uniform	Vacuolated	
**B4**	Regular petal	Uniform	Opaque unbroken or uniserial ladder	Medulla pattern not recognizable from cortex
**B5**	Double chevron	Uniform	Vacuolated	Cortex: more dense towards medulla
**B6**	Double chevron	Uniform	Vacuolated	Root shape: spade-like
				Cortex: more dense towards medulla
**B7**	Regular petal	Uniform	Vacuolated or uniserial ladder	Medulla pattern: wide

**Figure 3:**
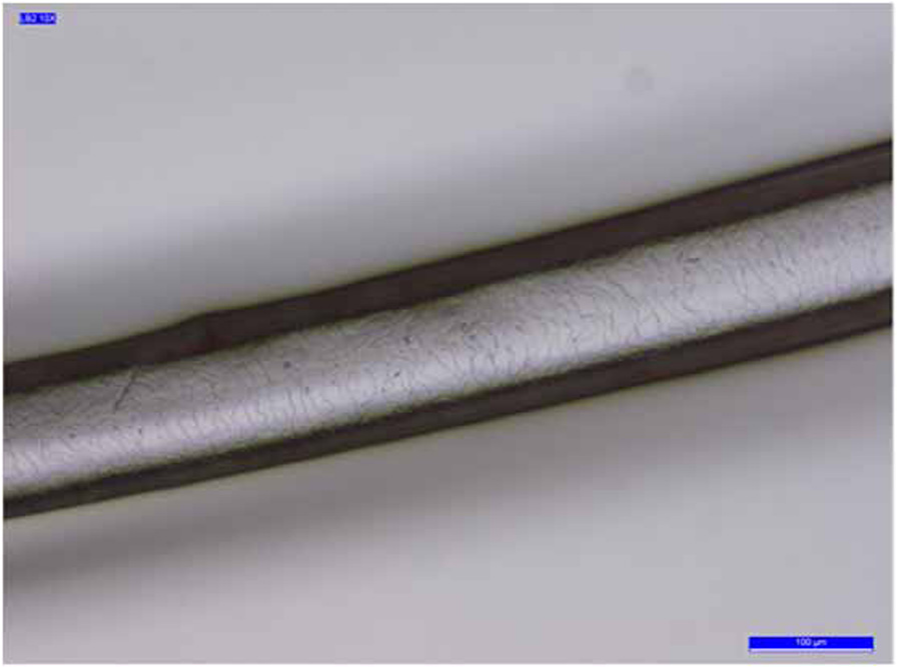
**Sample A3.** Example of the double-chevron scale pattern.

**Figure 4:**
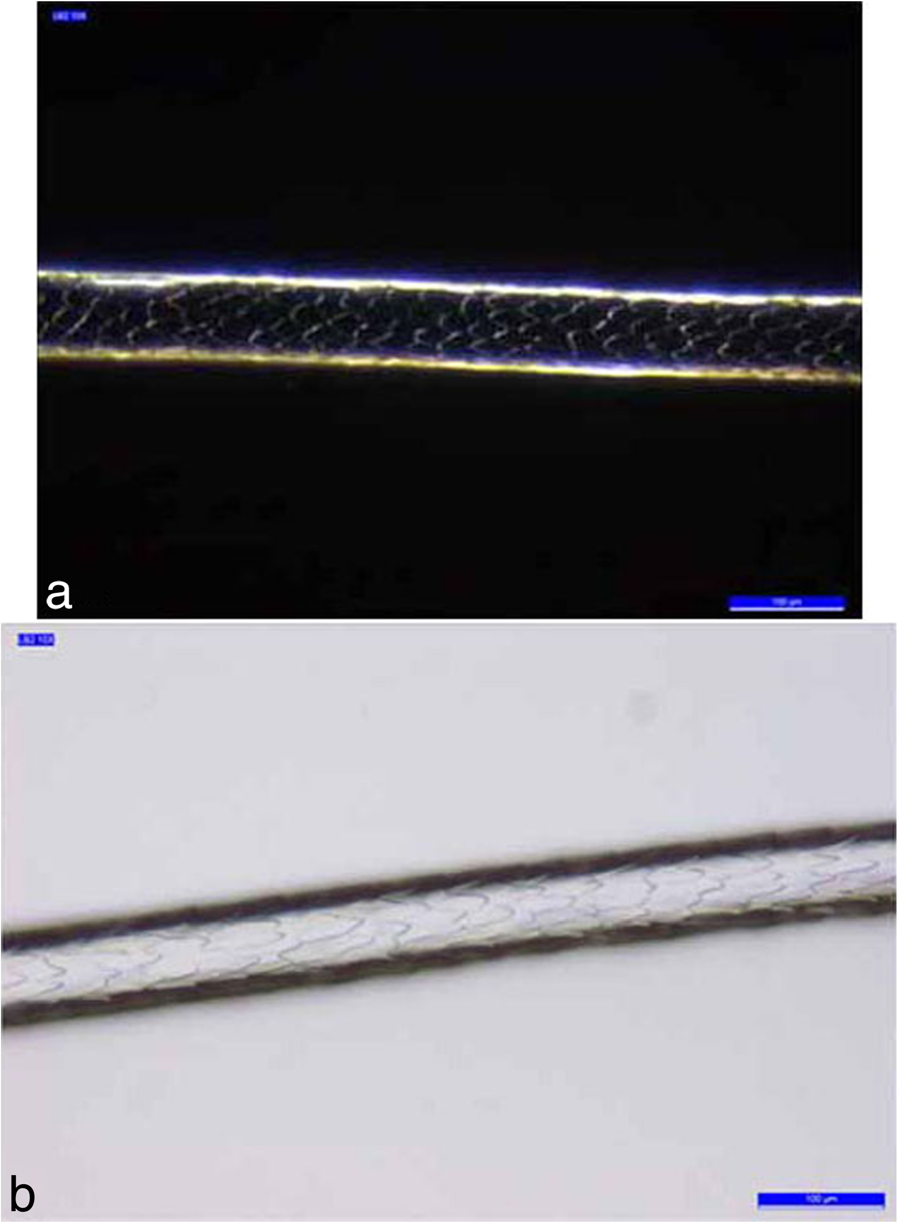
**Example of the regular petal scale pattern of the samples. (a)** B2 **(b)** B4.

The cuticle is a translucent layer of the hair shaft, consisting of scales covering the shaft. The observed cuticle scales ran from the root to the tip of the hair. In most samples, the structure of the scales was generally not prominent and showed an imbricate scale patterns (the double chevron of Table 3, shown in Figure 3); in others (B2, B4 -Figure 4- and B7) the scales were prominent and exhibited a spinous scale pattern (regular petal) that stuck out of the hair shaft and covered its entire length.

**Figure 5:**
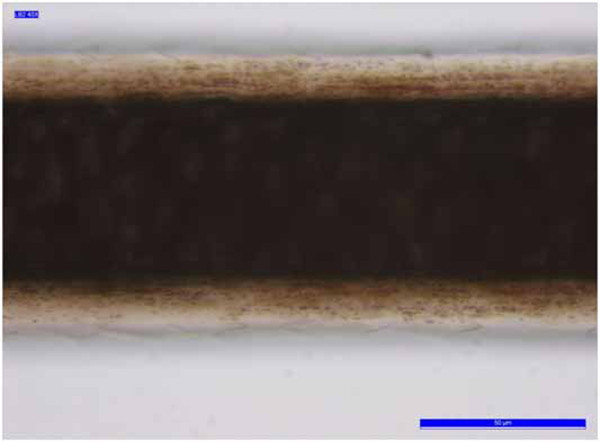
**Sample A3.** The cortex is more dense towards the medulla.

**Figure 6:**
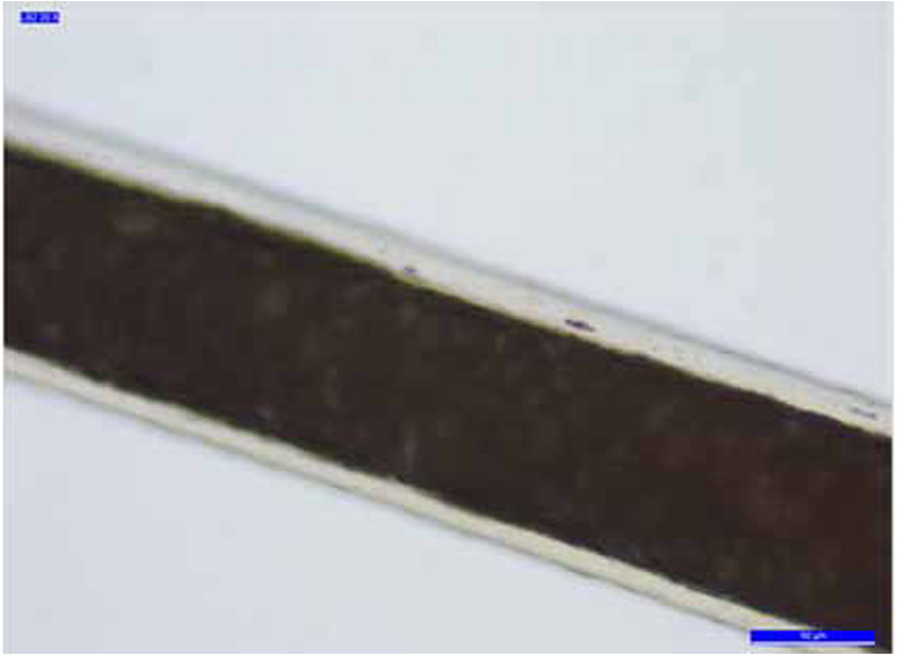
**Sample A2.** Example of cellular medulla pattern.

**Figure 7:**
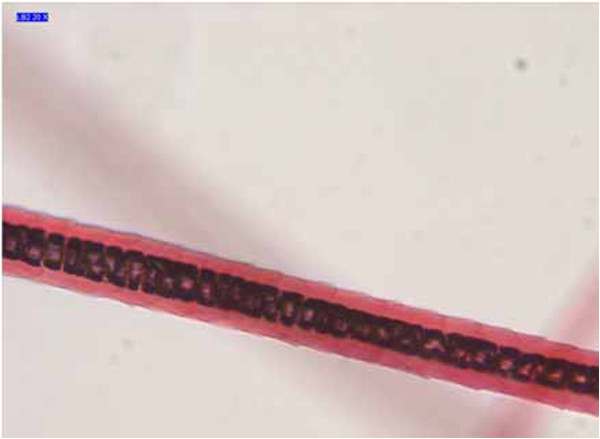
**Sample B1.** Example of vacuolated medulla pattern.

**Figure 8:**
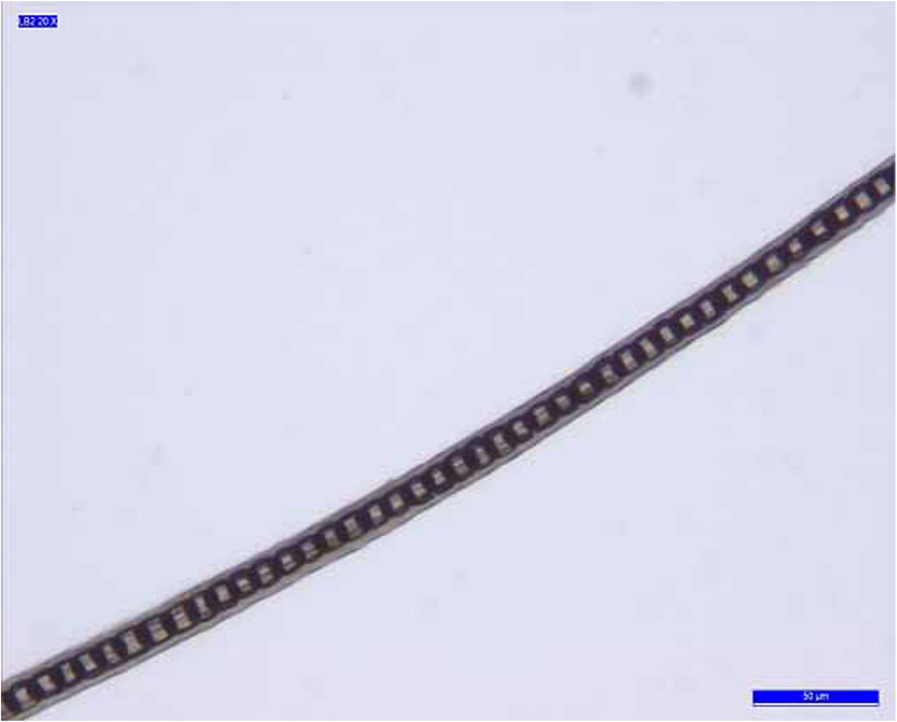
**Sample B7.** Example of uniserial ladder medulla pattern.

The nonprominent scale pattern is typical of dog hair, while the prominent scale pattern is typical of several animals, such as the sable, lynx, seal and cat. The cortex is the main body of the hair and is highly structured and organized. It is composed of elongated and fusiform cells with a pyknotic nucleus and fibrils that are the primary source of mechanical strength and water uptake. The cortical cells constitute the bulk of hair and the cortex provides the hair fibre with its eventual shape, resilience, elasticity and curl. In every sample, the cortex appeared uniform. However, in the cortex of some hair samples (A1, A3, A4, A5, B5 and B6, Table 3), an inhomogeneous pigmentation was noted along the length of the hair, mainly spread over the centre of the medullary region (Figure 5). The medulla is the innermost region of the hair, and is not always present. As with the other morphological characteristics, even the medulla can be a useful parameter to identify the species. Indeed both the diameter of the medulla and its structure are typical features of particular species. The medulla appeared continuous along the structure of the hair in every sample examined. In most samples, the medulla was cellular (Figure 6) or vacuolated (Figure 7) (the typical structure of a dog’s medulla) but in samples B2, B4 and B7 its structure was opaque unbroken and a uniserial ladder (Figure 8) (the representative configuration of a cat’s medulla).

In these samples, the medulla appeared wide. Generally, in animal hair, the medulla occupies an area greater than one-third of the overall diameter of the hair shaft and its value can change slightly among different species. Table 4 shows the medullary index of each sample (guard hair).

**Table 4:** Medullary diameter, overall diameter of hair shaft and medullary index of hair samples

**Samples**	**Medullary diameter (μm)**	**Overall diameter of hair shaft (μm)**	**Medullary index**
A1	94	122	0.77
A2	81	103	0.78
A3	56	85	0.66
A4	73	113	0.65
A5	38	54	0.70
B1	45	66	0.68
B2	42	64	0.68
B3	43	70	0.61
B4	Not detected	95	Not detected
B5	91	147	0.61
B6	51	85	0.60
B7	30	40	0.75

**Figure 9:**
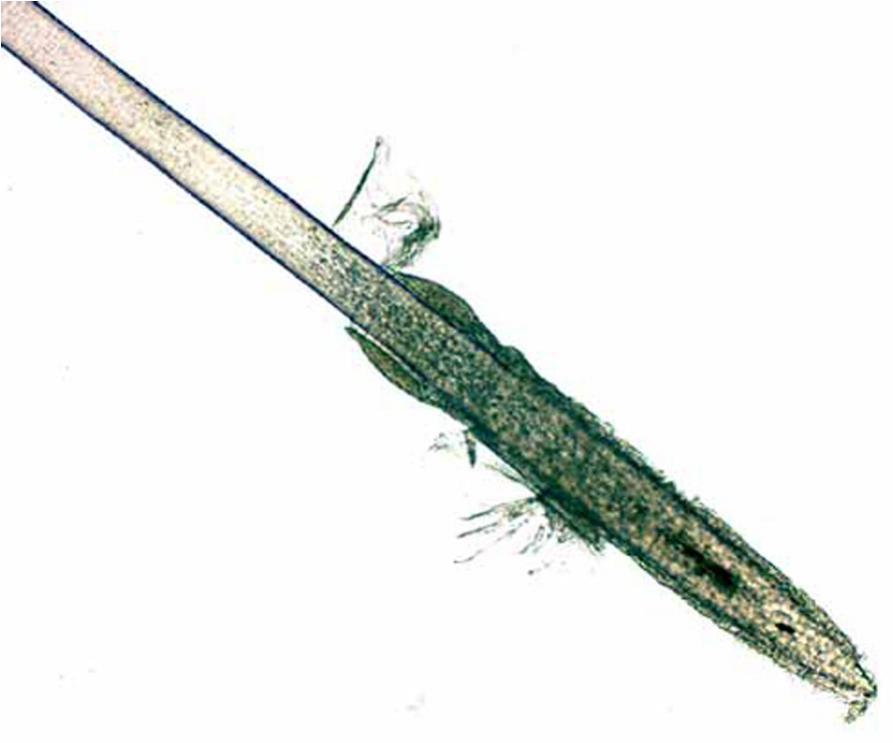
**Sample A2.** Example of spade-shaped root.

As can be deduced by the values reported in Table 4, the hair samples presented a fairly uniform distribution of the medullary index value of about 0.6 to 0.7; however, the index was never higher than 0.8 for any sample. These values were compatible with those of animal hair; indeed animals have a medullary index ranging from 0.5 to 0.9. However, despite the medullary index values being compatible with the Canidae and Felidae families, they were not indicative of any species in particular, since they ranged from half to three-quarters of the width, as is the case with the majority of species. In samples A2 and B6, microscopic analysis also showed the presence of spade-shaped roots. This is a typical characteristic of dog hair roots (Figure 9).

In summary, the furs appeared to be in good condition despite signs of physical and chemical stress due to tanning. These signs were particularly clear in the state of conservation of the underlying substrate and in the strong artificial dyeing of some hair. Microscopic inspection and evaluation of the medullary index [135] allowed us to establish the animal nature of the samples examined. The morphostructural data also provided an indication of the species to which the hair belonged, although they did not provide any kind of certainty about the specific species. Samples A1, A2, A3, A4, A5, B1, B3, B5 and B6 appeared as a homogeneous group with similar features, both macroscopic and microscopic, although macroscopically B1 and B3 showed intense artificial colouring different from the other fur samples. Therefore, on the basis of macro- and microscopic analysis, it was plausible to hypothesize that they shared a common origin, which could be ascribed to both the Canidae and the Procyonidae family. Yet, while the cuticle patterns (double chevron) and medulla patterns (vacuolated) are features of the Canidae family and the spade-like root shape of samples A2 and B6 is a typical structure in the hair roots of dogs, the medulla patterns (cellular), medullary indices and colour banding of the fur were more similar to a laboratory comparison sample from a raccoon [136]. Moreover, samples B2, B4 and B7 could be considered a separate group, with the same artificial colour of fur and comparable morphostructural characteristics consistent with a feline origin.

On the basis of the macroscopic and microscopic characteristics, it could be assumed that we were in the presence of hair belonging to the Canidae, Procyonidae or Felidae families. However, the identification of the family (Canidae or Procyonidae) and the species of these samples could not be confirmed by microscopic analysis, owing to the great variability of hair, the small amount of hair analyzed and the lack of a suitable comparative database or reference sample to compare the assay samples.

### Genetic and phylogenetic analysis

Owing to the chemical and physical damage [109,137-140] of the samples’ DNA during the processing cycle of tanning, PCR amplification of the *12S* and *16S* markers did not produce results in every sample analyzed. With regard to the *12S* marker, 10 samples out of 12 were successfully amplified, as shown in Table 5. For the *16S* marker, only 4 samples out of 12 were successfully amplified, probably as a result of the larger fragment size (244 bp, in contrast with the 150 bp of the *12S*), as shown in Table 6. The PCR products were cloned and 20 colonies were sequenced for each of them, to detect possible nucleotide misincorporations or contaminations. To identify the species, the consensus sequence produced for each sample was compared with the NCBI database on GenBank. A great many sequences are held on the GenBank database and even if a complete match cannot be found, a BLAST search allows identification of possibly closely related species, providing information at the level of the genus or family. The results obtained from this search and the percentage of similarity between the fur samples and the NCBI references for each marker analyzed are summarized in Table 5 and Table 6.

**Table 5:** **Results of *12S *** **marker analysis**

**Sample name**	***12S *** **consensus sequence**	**Identified species**	**Percentage similarity**
A1	✓	*Canis lupus laniger, Canis lupus chanco and Canis lupus familiaris*	98% match
A2	✓	*Canis lupus laniger, Canis lupus chanco and Canis lupus familiaris*	98% match
A3	✓	*Canis lupus laniger, Canis lupus chanco and Canis lupus familiaris*	98% match
A4	✓	*Canis lupus laniger, Canis lupus chanco and Canis lupus familiaris*	98% match
A5	✓	*Homo sapiens*	93% match
B1	✓	*Homo sapiens*	93% match
B2	✓	*Homo sapiens*	93% match
B3	✓	*Homo sapiens*	93% match
B4	=	Not available	
B5	=	Not available	
B6	✓	*Canis lupus laniger, Canis lupus chanco and Canis lupus familiaris*	98% match
B7	✓	*Felis silvestris catus*	100% match

Species identification of fur samples and percentage of similarity after research conducted using the BLAST tool. ✓ success in obtaining the consensus sequence; = failure in obtaining the consensus sequence.

**Table 6:** **Results of *16S *** **marker analysis**

**Sample name**	***16S *** **consensus sequence**	**Identified species**	**Percentage of similarity**
A1	✓	*Canis lupus familiaris*	99% match
A2	✓	*Canis lupus familiaris*	99% match
A3	✓	*Homo sapiens*	95% match
A4	=	Not available	
A5	=	Not available	
B1	=	Not available	
B2	✓	*Homo sapiens*	95% match
B3	=	Not available	
B4	=	Not available	
B5	=	Not available	
B6	=	Not available	
B7	=	Not available	

Species identification of fur samples and percentage of similarity after research conducted using the BLAST tool. ✓ success in obtaining the consensus sequence; = failure in obtaining the consensus sequence.

**Figure 10:**
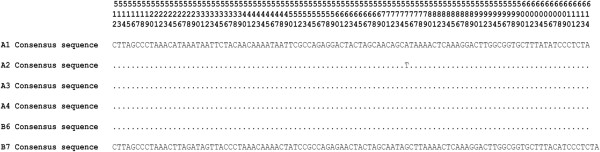
***12S *consensus sequences of fur samples.** Alignments of the *12S* consensus sequences of fur samples (A1, A2, A3, A4, B6 and B7). Nucleotide positions are numbered according to GenBank GU256221.1 [141].

Probably as a result of contamination by human genetic material from those who treated or handled the furs, which was not entirely eliminated during the cleaning of the samples, the consensus sequence obtained from samples A5, B1, B2 and B3 (*12S* marker) and A3 and B2 (*16S* marker) showed a 93 to 95% degree of similarity to the reference sequence for *Homo sapiens*. A match with 100% of homology between the short *12S* fragment (150 bp) from the B7 sequence (Figure 10) and the NCBI reference of *Felis silvestris catus* was observed. This complete degree of homology could be explained as follows: either the sample comes from the species with which it matches or, because of the shortness of the sequence fragment, the sample matches that NCBI reference by chance and comes from an unknown species in GenBank. Furthermore, a search on GenBank for a comparison with sequences belonging to different *Felis* species or subspecies returned results only for the domestic species concerning this genetic marker. Owing to the availability of this short DNA sequence and the lack of knowledge about the variability between different species or subspecies for the *12S* fragment, no phylogenetic analysis was performed for this sample and the genus *Felis*. For these reasons, it was not possible to attribute the hair solely to the domestic subspecies.

**Figure 11:**
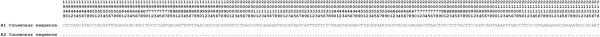
***16S *consensus sequence of fur samples.** Alignments of the *16S* consensus sequences of fur samples (A1 and A2). Nucleotide positions are numbered according to GenBank GU256221.1 [141].

The *12S* consensus sequences of samples A1, A3, A4 and B6 (Figure 10) were identical to each other, as was the sequence for the *16S* in samples A1 and A2 (Figure 11), presumably because the hair analyzed belonged to the same species. Only the *12S* consensus sequence of sample A2 showed a transversion (T instead of A) when compared with the other samples analyzed (Figure 10). The 98% homology between these sequences and NCBI references of *Canis lupus laniger*, *Canis lupus chanco* and *Canis lupus familiaris* was observed and could be explained in two ways. Either the unknown samples came from one of these species and the differences were due to intraspecific variation, or they came from an unknown but closely related species that was not present on the database.

**Figure 12:**
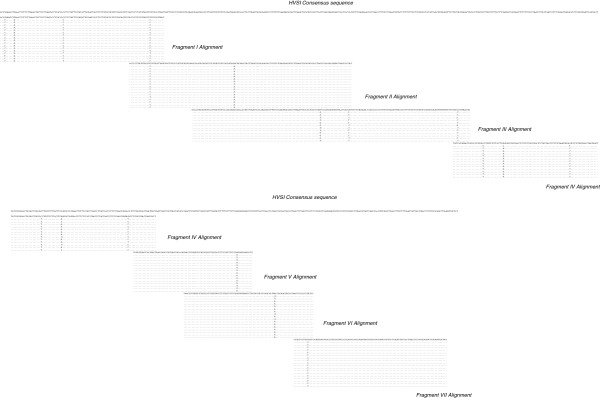
**Alignment of HVS-I amplicons from the cloning of sample A2.** DNA sequences from the clones analyzed for the A2 sample are reported as an example. Nucleotides identical to the A2 sequence are indicated by dots. Some misincorporations can be observed in some clones.

**Figure 13:**
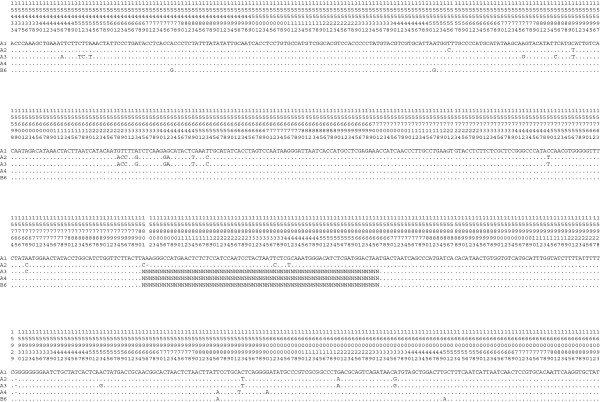
**HVS-I consensus sequences of fur samples.** Alignments of the HVS-I consensus sequences of fur samples (A1, A2, A3, A4 and B6). The numbering of the nucleotide positions is at the top, according to GenBank GU256221.1 [141]. Nucleotides identical to the A1 sequence are indicated by dots.

To obtain an unambiguous attribution of the hair to the subspecies listed, and distinguish the fur samples from potential different individuals, the analysis focused on the study of the HVS-I of the canine D-loop. The amplification of HVS-I using seven overlapping fragments (Figure 12) led to a complete consensus sequence for samples A1 and A2. For samples A3, A4 and B6, the amplification of the IV fragment (150 bp) failed, maybe because of degradation phenomena with possible modification in the annealing site of the primers. However, it is most likely that the failure to obtain a result could be explained by the presence of mutations in the DNA template that prevented the annealing of one or both of the two primers. The complete consensus sequences of A1 and A2 samples were aligned with each other and with the partial consensus sequences of samples A3, A4 and B6. All consensus sequences are available at the National Center for Biotechnology [GeneBank Accession Numbers: KJ828711-KJ828715]. The sequences were similar but not the same (Figure 13). Owing to the large variability of the genetic region analyzed, we presumed that the hair could have belonged to different individuals. Both the complete consensus sequences of samples A1 and A2 and the partial ones of A3, A4 and B6 were compared with those held on GenBank. The results obtained showed a homology of 97% to 99% with *Nyctereutes procyonoides*. The degree of match was not 100% and this could be explained in the following ways: (1) different individuals of the same species could have different genetic profiles because the marker analyzed was a highly variable region; (2) some of the differences observed between unknown and reference samples were the result of *post mortem* damage [112,142,143], that is, the modifications in DNA sequence arose subsequent to cell death or as a result of the tanning process. The apparent inconsistency found when analyzing the results of mtDNA (*12S*, *16S* and HVS-I) can be explained by the small amount of data available in the literature on the genome of *Nyctereutes procyonoides* and, at the time of the realization of this work, by the absence of the *12S* sequence of this species in the NCBI database. Therefore, the most likely diagnosis of the species was that of the *Nyctereutes procyonoides*, the raccoon dog. This conclusion was confirmed when the *12S* sequence of the *Nyctereutes procyonoides* genome was published in GenBank.

**Figure 14:**
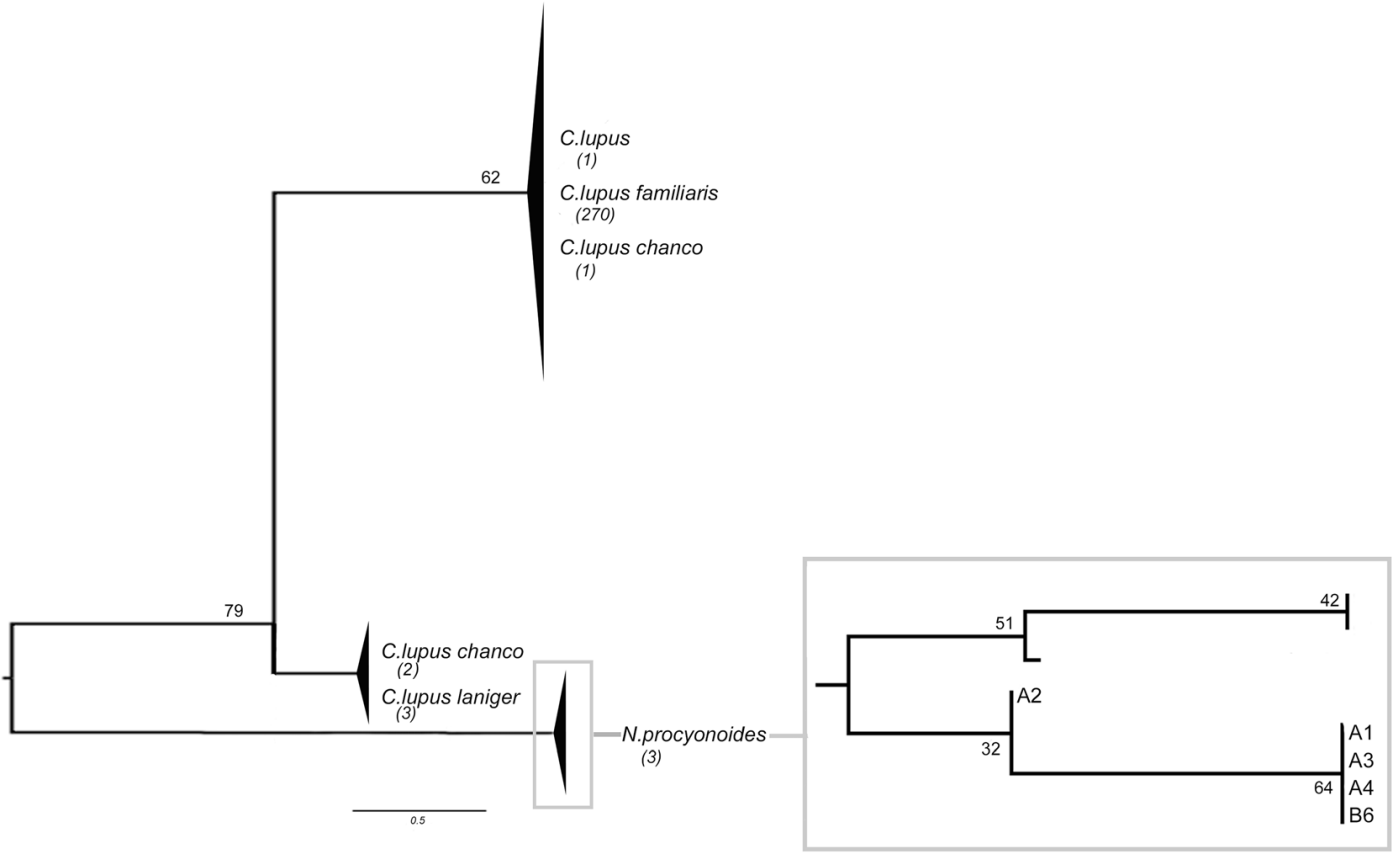
**Neighbour-joining tree built for *12S *marker.** The bootstrap neighbour-joining tree inferred from 1,000 replicates represents the evolutionary history of 285 sequences (280 NCBI reference sequences and five fur samples) of the *12S* marker. The reference sequences were retrieved from the species that presented almost 98% of similarity to the sequences obtained from fur samples A1, A2, A3, A4 and B6. The bootstrap percentage values are indicated at the nodes. In each clade, the number of comparison sequences is indicated below the name of the species. The *Nyctereutes procyonoides* clade is enlarged for better visibility within the grey box.

**Figure 15:**
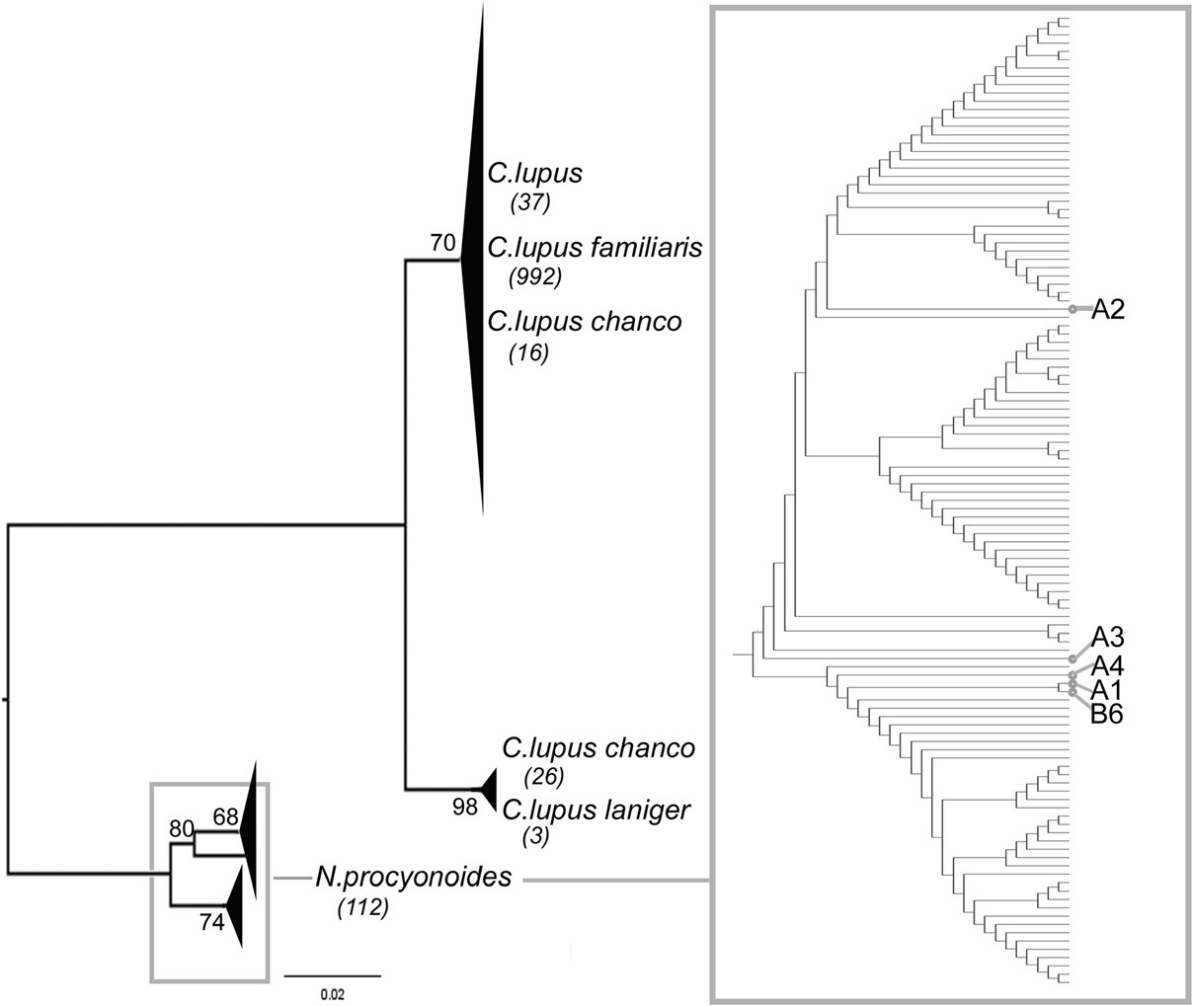
**Neighbour-joining tree built for HVS-I.** The bootstrap neighbour-joining tree inferred from 1,000 replicates represents the evolutionary history of 1,191 sequences (1,186 NCBI reference sequences and five fur samples) of the HVS-I marker. The reference sequences were retrieved from the species that presented almost 98% of similarity to the sequences obtained from fur samples A1, A2, A3, A4 and B6. The bootstrap percentage values are indicated at the nodes whenever larger than 50. In each clade, the number of comparison sequences is indicated below the name of the species. The *Nyctereutes procyonoides* clade is enlarged for better visibility within the grey box. This subtree is condensed for bootstrap values lower than 50 and only topology is shown. The position of the fur samples is indicated by grey dots linked to the name of the sample.

The next comparison of the *12S* consensus sequence of samples A1, A2, A3, A4 and B6 with those held on GenBank showed the highest homology (100%) with *Nyctereutes procyonoides* for samples A1, A3, A4 and B6 and 99% homology with the same species for sample A2. The next comparison of the consensus sequence for *16S* of samples A1 and A2 showed the highest homology (100%) with *Nyctereutes procyonoides*. These data confirmed the information obtained from the HVS-I. Several sequences (280 for the *12S* marker and 1,186 for the HVS-I) from the NCBI database relative to all species or subspecies that showed a homology of almost 98% with fur samples were collected in a dataset (Table 7). To better investigate how the fur samples fell into the genetic variability of the comparison dataset, phylogenetic analyses were performed. The neighbour-joining tree for the *12S* marker (Figure 14) was calculated using the Kimura two-parameter model [144]: this described the substitution pattern in the dataset of sequences better than other available models. Genera *Canis* and *Nyctereutes* were separated into two groups with a bootstrap value of 79%. The three available sequences of *Canis lupus chanco* were distributed as follows: one in the subgroup of *Canis lupus* and *Canis lupus familiaris*, and the others in that of *Canis lupus laniger*. All fur samples fell into the same group of *Nyctereutes procyonoides* but the three NCBI reference sequences differed from ours. The neighbour-joining tree obtained from the alignment between the HVS-I consensus sequences of the fur samples and the comparison dataset is shown in Figure 15. The tree was calculated using the Tamura three-parameter model of pairwise base substitution [145]. This was the best model to describe sequence divergence in the dataset. Overall groupings of the sequences in the dataset were as expected: genera *Canis* and *Nyctereutes* were separated into two groups, with the maximum value of statistical support. A 100% bootstrap value at the node connecting these genera indicated that the genetic marker (HVS-I) we used had enough informative power to make the two groups distinguishable.

**Table 7:** Number of sequences and their origin for the dataset used in the phylogenetic analysis

**Species**	**Number of sequences**	**GenBank accession numbers**
*Canis lupus*	37	AF005296-005314,
		AF008135-008142,
		AF008160-008167 [146]
		AY240073, AY240155 [125]
*Canis lupus familiaris*	1,111	NC002008, U96639* [127]
		EU223385-223811 [147]
		AY656703-656710 [148]
		AY656737-656755* [149]
		EU816456-816557 [150]
		AF531654-531741,
		HQ261490 [151]
		AF005280-005295,
		AF008143-008157,
		AF008168-008182 [146]
		JF342807-342906 * [152]
		AY240030-240072,
		AY240074-240093,
		AY240095-240154,
		AY2400156-2400157 [125]
		AF064569-064586 [153]
		EF122413-122428 [94]
		AB622516-622568,
		AB700664-700665 [154]
		EU789667-789786** [150]
*Canis lupus laniger*	3	KF573616* [155]
		NC011218, FJ032363* [156]
*Canis lupus chanco*	42	AB007375-007379 [157]
		AB480743-480744 [158]
		AY333738-333742 [159]
		EU442884* [160]
		GQ374438* [141]
		JX415343-415379 [161]
		NC010340* [156]
*Nyctereutes procyonoides*	113	AB292740 [162]
		D83614-83615 [163]
		EU642411-642457 [164]
		FJ888513-888521 [165]
		JF809819-809848 [166]
		KC344215-344235,
		KC509604 [167]
		GU256221* [141]
		NC013700* [168]
		KF781340** [169]

All sequences reported in the table were used for HVS-I comparison. Those marked with * were used both for HVS-I and *12S* and those marked with ** were used only for *12S* comparison.

In the genus *Canis*, some sequences of *Canis lupus chanco* cluster together with *Canis lupus* and *Canis lupus familiaris*, while others belong to the same group of *Canis lupus laniger.* Consensus sequences of the fur samples fell clearly into the variability of the *Nyctereutes procyonoides* group. The phylogenetic analysis confirmed the indications given by the BLAST search, showing samples A1, A2, A3, A4 and B6 as belonging to the *Nyctereutes procyonoides* species. Although *Nyctereutes procyonoides* is often confused with raccoons and badgers, it does not belong to the Procyonidae family but to the Canidae, as does the domestic dog. *Nyctereutes procyonoides* and the domestic dog present the genus they belong to: the raccoon dog actually belongs to the genus *Nyctereutes,* while the domestic dog belongs to the genus *Canis*. The physical characteristics of the raccoon dog are substantially similar to those of a medium-sized domestic dog with the indicative feature of a raccoon snout, although it is distinguished taxonomically from the raccoon itself. *Nyctereutes* comes from Japan, Siberia and Manchuria.

## Conclusions

Macroscopic and microscopic analysis allowed confirmation of the hypothesis regarding the analyzed hair belonging to real animals, although it failed to provide any kind of certainty regarding the actual family or species. The sequence data and the comparisons with the samples held on GenBank showed that, in most cases, the hair belonged to the *Canidae* family, and in only one case to *Felidae*. The genetic data related to the diagnosis of the genus and the species of *Canidae* hair was heterogeneous: the sequence results of samples A1, A2, A3, A4 and B6 (*12S*, *16S* and HVS-I) showed a high homology with the genus *Canis* and the genus *Nyctereutes* (raccoon dog). This initial uncertainty led obviously to a likely ambiguous diagnosis of the species that was impossible to predict. However, the genetic results confirmed a unique diagnosis of nonattribution for the protected subspecies *Canis lupus familiaris*. Despite previous reservations advanced, the species that appeared more consistent with the data obtained from the BLAST search was *Nyctereutes procyonoides*, and phylogenetic analysis confirmed this observation*.*

The data of the diagnosis of the genus and the species of the hair belonging to the *Felidae* family appeared homogeneous. The study revealed a high similarity between our sequences and the genus *Felis*, species *Felis silvestris*. However, the failure to diagnose a single domestic subspecies (*Felis silvestris catus*) was mainly due to the limited amount of genetic data for comparative purposes in the literature. In fact, no information on the genetic differences between the various subspecies and on the process of cat domestication [170,171] are known and no genetic data are available on GenBank for different subspecies of *Felis silvestris* regarding the *12S* marker. For these reasons, an unambiguous attribution of one of the fur samples to the subspecies *Felis silvestris catus* was not possible. Finally, we would like to add that beyond scientific classification, prohibition of the use for commercial purposes should not be just confined to the subspecies *Canis lupus familiaris or Felis silvestris catus* but should be extended to other Canidae and Felidae, particularly when taking into account sociocultural practices and geographical regions far from the West.

## Abbreviations

BLAST: Basic Local Alignment Search Tool; bp: base pair; *COI*: *cytochrome c oxidase subunit 1*; dNTP: deoxy-nucleotide-triphosphate; HVS: hypervariable segment; HVS-I: hypervariable segment I; HVS-II: hypervariable segment II; mtDNA: mitochondrial DNA; NDH: nicotinamide adenine dinucleotide; nt: nucleotide; PCR: polymerase chain reaction; SDS: sodium dodecyl sulphate; VNTR: variable number tandem repeat.

## Competing interests

The authors declare that they have no competing interests.

## Authors’ contributions

EP conceived and designed the experiments. EP, RC, AV and GD performed the experiments. EP, RC and SV analyzed the data. FB, AB, GL and DC contributed reagents, materials and analysis tools. EP wrote the paper. All authors read and approved the final manuscript.
